# Peri-Implantitis—An Overview of Treatment Options and a New Approach to the Treatment of Peri-Implantitis Using a Magnesium Membrane in Three Case Reports

**DOI:** 10.3390/dj14020112

**Published:** 2026-02-13

**Authors:** Domagoj Vražić, Katarina Komar Milas, Marko Blašković, Ivana Butorac Prpić, Marija Čandrlić, Željka Perić Kačarević

**Affiliations:** 1 Department of Periodontology, School of Dental Medicine, University of Zagreb, 10000 Zagreb, Croatia; vrazic@sfzg.hr; 2 Interdisciplinary University Study of Molecular Biosciences, Josip Juraj Strossmayer University of Osijek, 31000 Osijek, Croatia; katarina.komar@gmail.com (K.K.M.); butoracivana88@gmail.com (I.B.P.); 3 Department of Oral Surgery, Faculty of Dental Medicine Rijeka, University of Rijeka, 51000 Rijeka, Croatia; marko_blaskovic@yahoo.com; 4 Department of Dental Medicine, Faculty of Dental Medicine and Health Osijek, Josip Juraj Strossmayer University of Osijek, 31000 Osijek, Croatia; marija.candrlic@fdmz.hr; 5 Department of Anatomy, Histology, Embriology, Pathology Anatomy and Pathology Histology, Faculty of Dental Medicine and Health Osijek, Josip Juraj Strossmayer University of Osijek, 31000 Osijek, Croatia

**Keywords:** dental implants, peri-implantitis, magnesium membrane, bovine xenograft, hyaluronate, shield technique

## Abstract

**Background/Objectives:** Dental implants are a preferred solution for missing teeth, but peri-implantitis remains a major challenge in implant dentistry. This narrative review provides an overview of the therapeutic interventions for peri-implantitis based on the current literature and illustrates a new clinical approach using novel magnesium membrane through three case presentations. **Methods:** A comprehensive literature search on peri-implantitis management was conducted, with emphasis on current clinical practice guidelines. In addition, three clinical cases were presented to demonstrate the use of a fully resorbable magnesium membrane in combination with a bovine xenograft with hyaluronate. **Results:** The narrative review identified and summarized a wide range of non-surgical and surgical therapeutic strategies for treatment of peri-implantitis. Additionally, three case reports with novel magnesium membrane highlighted distinct clinical scenarios: (1) bone defect reconstruction without implant removal, (2) reconstruction following implant removal, and (3) a minimally invasive shield technique performed without removal of the implant or crown. All cases demonstrated favorable clinical outcomes following the novel biomaterial approach. **Conclusions:** The combination of a resorbable magnesium membrane with bovine xenograft with hyaluronate represents a promising therapeutic strategy for treatment of peri-implantitis. This approach may improve clinical outcomes and potentially set new standards in implant dentistry. Further studies with larger cohorts and control groups are required to confirm these preliminary findings.

## 1. Introduction

Dental implants represent a predictable and widely used solution for the replacement of missing teeth, with high survival rates reported in long-term follow-up studies [1,2].

Despite their overall success, biological complications remain a significant challenge in daily clinical practice, with peri-implantitis being the most severe condition affecting implant-supported rehabilitations [3,4]. If left untreated, peri-implantitis leads to progressive peri-implant bone loss and may ultimately result in implant failure [5,6]. Peri-implantitis is a plaque-associated pathological condition characterized by inflammation of the peri-implant mucosa and progressive loss of supporting bone [7]. Clinically, it is diagnosed by bleeding and/or suppuration on probing, increased probing depth, and radiographic bone loss beyond initial remodeling [8]. Its etiology is multifactorial and involves microbial colonization, host immune response, and local and systemic risk factors. A history of periodontitis, inadequate oral hygiene, and insufficient supportive peri-implant care are among the most consistently reported risk factors [9,10]. Compared with periodontitis, peri-implantitis is often associated with a more aggressive inflammatory infiltrate and faster circumferential bone destruction, which limits the capacity of peri-implant tissues to self-contain disease progression [11,12]. Microbiological studies have demonstrated that peri-implantitis is associated with a biofilm dominated by anaerobic Gram-negative bacteria commonly implicated in periodontitis, including *Porphyromonas gingivalis*, *Tannerella forsythia*, and *Treponema denticola* [13,14]. In addition, peri-implantitis lesions exhibit an altered host immune response, with increased expression of proinflammatory mediators such as IL-1β, IL-6, IL-8, TNF-α, and IL-17 compared with healthy peri-implant tissues [15].

The primary goal of peri-implantitis therapy is the elimination of the bacterial biofilm and the control of inflammation. Depending on disease severity, defect morphology, and patient-related factors, treatment may range from non-surgical approaches to surgical resective or regenerative procedures [16,17]. While non-surgical therapy plays an essential role in initial infection control [18], surgical intervention is frequently required in advanced cases to allow access to contaminated implant surfaces and reconstruction of peri-implant bone defects [19].

Regenerative surgical approaches aim to restore lost peri-implant bone and promote re-osseointegration of the affected implant surface [20]. Guided bone regeneration (GBR) using bone substitute materials and barrier membranes is widely applied [21,22]; however, conventional membranes present limitations related to mechanical stability, degradation behavior, and susceptibility to exposure in inflammatory environments [23,24]. Recently, resorbable magnesium membranes have emerged as a promising alternative due to their favorable mechanical properties, biodegradability, and bioactive degradation products that may support osteogenesis and modulate bacterial adhesion [25].

The aim of this narrative review is to summarize current therapeutic strategies for peri-implantitis with a focus on contemporary clinical practice guidelines and regenerative concepts. In addition, three clinical cases are presented to illustrate a novel regenerative approach using a fully resorbable magnesium membrane for the treatment of peri-implantitis.

## 2. Methodology

### 2.1. Literature Search

A comprehensive literature search was conducted up to December 2025 using PubMed/MEDLINE, Scopus, and Web of Science databases. The search strategy combined the following keywords and MeSH terms: “peri-implantitis,” “dental implants,” “peri-implant disease,” “risk factors,” “treatment,” and “management”. Synonyms within the same concept were combined using the Boolean operator OR, while different concepts were combined using AND.

Studies of various designs, including randomized controlled trials, cohort studies, systematic and narrative reviews, as well as relevant in vitro and animal studies, were included to provide a overview of therapeutic strategies and to describe the biological and mechanical characteristics of magnesium membranes. The search was limited to articles published in English. Articles were screened based on relevance to peri-implantitis treatment modalities, including non-surgical and surgical approaches, adjunctive therapies, and novel regenerative techniques. As this is a narrative review, evidence synthesis was descriptive rather than quantitative.

### 2.2. Case Reports—General Methodology

Ethical approval for the clinical and radiographic procedures was obtained from the Ethics Committee of the Faculty of Dental Medicine and Health Osijek, University J.J. Strossmayer of Osijek (Class: 602-01/23-12/03, No: 2158/97-97-10-23-22, date of approval: 6 April 2023), and the clinical treatment started in June 2023. All procedures were conducted in accordance with the Declaration of Helsinki.

Three patients presenting with clinical and radiographic signs of peri-implantitis were consecutively selected from a private dental practice based on predefined inclusion and exclusion criteria. Inclusion criteria comprised age ≥18 years, absence of allergies, non-smoking status, good oral hygiene, and provision of written informed consent. Exclusion criteria included uncontrolled or severe systemic diseases, medications affecting bone metabolism or wound healing, prior head and neck radiotherapy, and pregnancy or lactation. Treatment decisions, including implant retention or removal, were made on a case-by-case basis according to defect morphology, implant integrity, prosthetic considerations, and patient-related factors.

Each patient was adequately informed about the purpose of the treatment protocol and possible complications. The surgeries were performed by two experienced surgeons (M.B. and D.V.), after each patient signed the informed consent form.

All patients underwent an initial non-surgical treatment phase prior to surgery, aimed at reducing peri-implant inflammation and improving plaque control. This phase included full-mouth scaling and ultrasonic polishing with tips softer than titanium (i.e., plastic and Teflon-coated tips), individualized oral hygiene instructions, and clinical and radiographic assessment using orthopantomography and cone beam computed tomography (CBCT) with a three-dimensional imaging system (ProMax 3D, Planmeca Oy, Helsinki, Finland). After a re-evaluation period of approximately six weeks, clinical improvement was observed in all cases; however, due to the persistence of residual peri-implant bone defects, surgical intervention was indicated. Following the surgical procedures, systemic antibiotic therapy was administered (Amoksicilin 1000 mg, Belupo, Koprivnica, Croatia). Patients received postoperative oral hygiene instructions and were enrolled in a recall program that included clinical and radiographic outcome assessments during the subsequent months.

## 3. Case Reports

### 3.1. Case Report 1—Surgical Treatment of Peri-Implantitis Without Implant Removal

**Figure 1:**
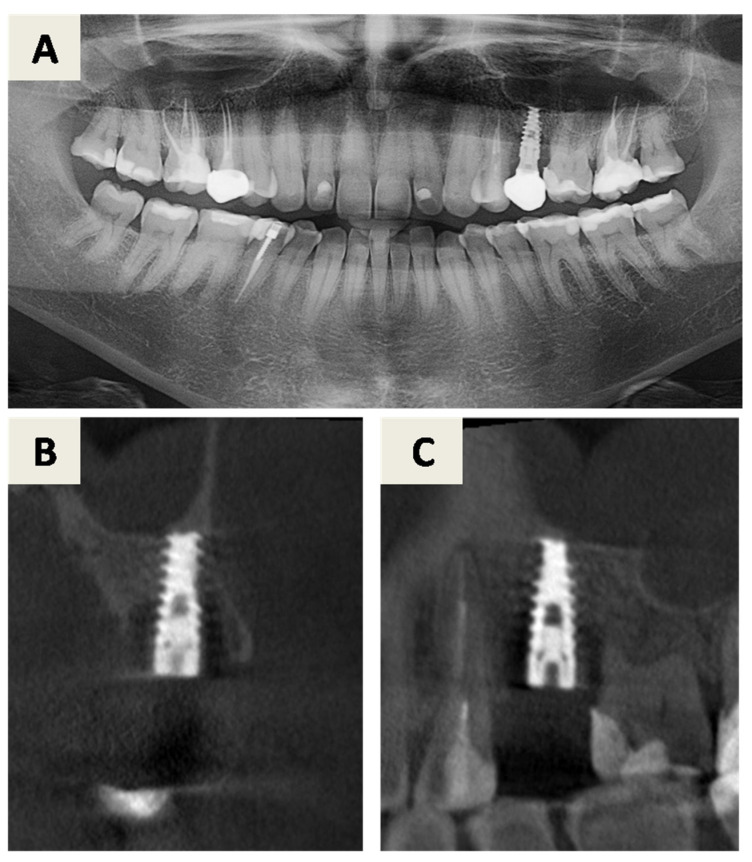
First radiographic examination. (**A**) Orthopantomogram showing visible peri-implantitis related bone defect around the implant in position 25 (FDI notation system). (**B**,**C**) Cone beam computed tomography (CBCT) images in coronal (**B**) and sagittal (**C**) planes taken 6 weeks after crown removal showing visible defect of the palatal bony surface extending to ½ length of the implant and a small (2–3 mm) supracrestal defect located around the implant shoulder.

**Figure 2:**
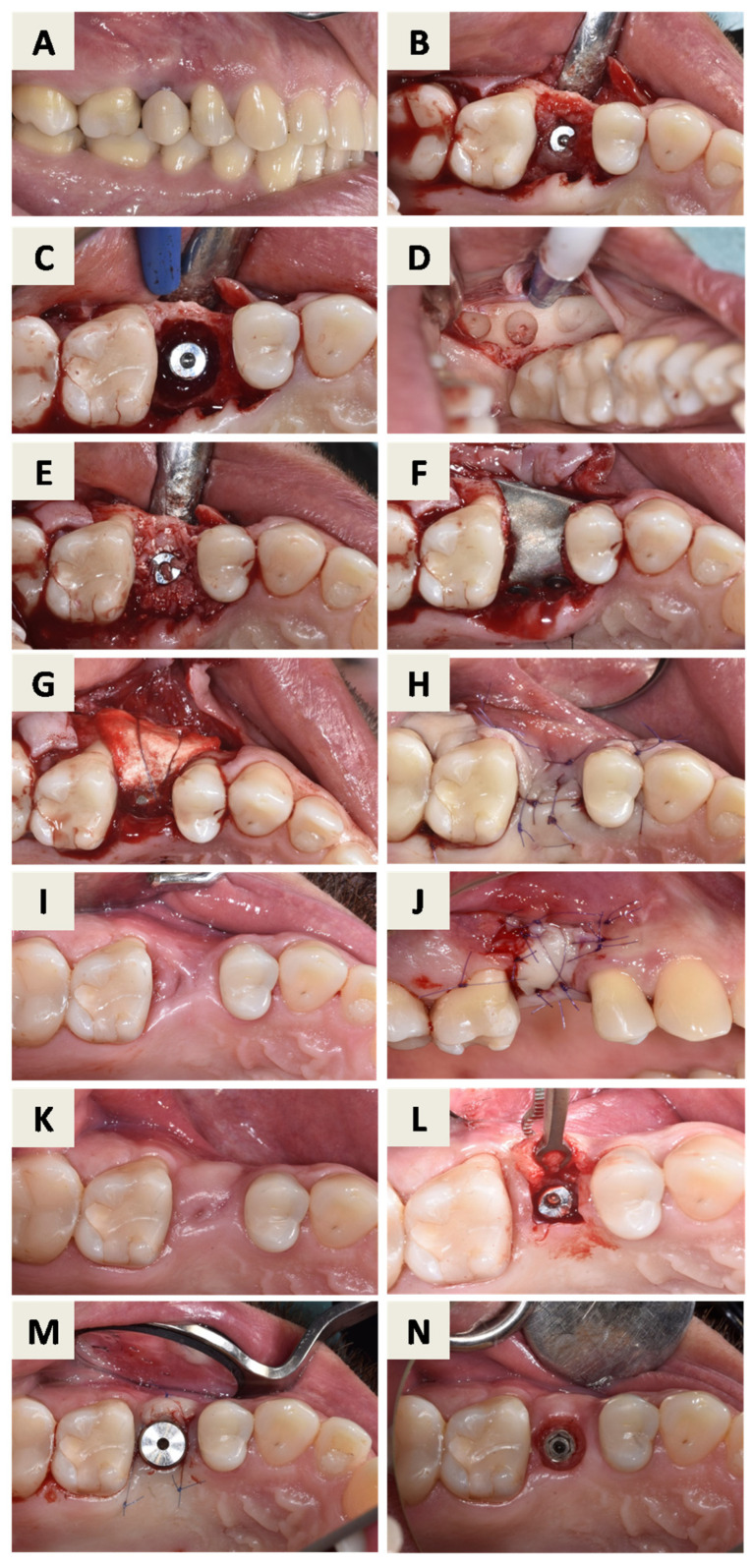
Surgical procedure: (**A**) Clinical situation before removal of the inadequate crown at implant position 25. After removal of the crown, the cover screw was placed on the implant and the soft tissue was able to heal spontaneously. (**B**) 6 weeks after removal of the crown, a full thickness flap was elevated showing granulation tissue around the affected implant. (**C**) After removal of the granulation tissue, a combined bone defect can be seen around the implant. (**D**) Harvesting of autologous bone from the left retromolar area. (**E**) The mixture of autologous bone graft particles and previously hydrated particles of bovine bone with hyaluronate was placed around the implant. (**F**) The magnesium membrane was cut to size and adapted to the individual shape of the defect. The membrane was fixed with suitable resorptive magnesium screws. (**G**) Pericardium collagen membrane was placed on the magnesium membrane and fixed with sutures. (**H**) The flap was closed and sutured to heal with primary intention. (**I**) Site after 4 months, visible lack of keratinized mucosa width, reduced soft tissue thickness and soft tissue defect mesial to tooth 26. (**J**) Free gingival graft procedure, stabilized with sutures. (**K**) Healed site after 6 months. (**L**) Implant uncovering procedure. (**M**) Primary wound closure around healing abutment (**N**) Healed peri-implant soft tissue transition zone after 4 weeks.

The treatment plan for peri-implantitis at implant site 25 (FDI Notation System) (Figure 1) consisted of a regenerative approach following removal of the inadequate implant crown. Despite advanced bone loss, the implant was retained due to the absence of implant mobility, a predominantly contained defect morphology suitable for regeneration, and its strategic prosthetic importance. Pre-surgical assessment included evaluation of orthopantomogram and CBCT images (Figure 1) as well as clinical parameters (Figure 2A). At this stage, the implant-supported crown was removed, a cover screw was placed, and spontaneous soft tissue healing was allowed.

After mouth rinsing with a chlorhexidine digluconate solution (Parodontax^®^ 0.2%, Brentford, London, UK), a local anesthesia with 4% articaine and epinephrine 1:100,000 (Ubistesin^®^ Forte 40 mg/mL +0.01 mg/mL, 3M Deutschland GmbH, Seefeld, Germany) was administered. An intrasulcular incision with one vertical releasing incision at the mesial surface of tooth 24 was performed, and a full-thickness flap was elevated, revealing granulation tissue surrounding the implant (Figure 2B). Granulation tissue was carefully removed, demonstrating a supracrestal defect around the affected implant (Figure 2C). Implant surface decontamination was then performed by mechanical debridement using titanium curettes and copious irrigation with sterile saline solution.

During the same surgical session, autologous bone was harvested from the left retromolar area (Figure 2D). The harvested bone particles were mixed with hydrated bovine xenograft with hyaluronate (cerabone^®^ plus, botiss biomaterials GmbH, Berlin, Germany) and placed into the peri-implant defect (Figure 2E). The magnesium membrane (NOVAMag^®^ membrane, botiss biomaterials GmbH, Berlin, Germany) was cut to size and adapted to the individual shape of the defect, after which it was fixated on the buccal and palatal surface of the alveolar bone, apical from the defect with suitable resorptive magnesium screws (NOVAMag^®^ fixation screw XS, botiss biomaterials GmbH, Berlin, Germany) (Figure 2F). A pericardium collagen membrane (jason^®^ membrane, botiss biomaterials GmbH, Zossen, Germany) was placed over the magnesium membrane for augmentation and soft tissue profiling and secured with resorbable sutures (Figure 2G). Primary wound closure was achieved (Figure 2H).

**Figure 3:**
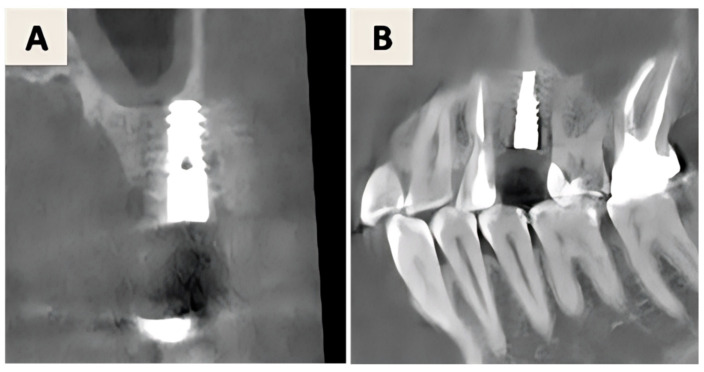
CBCT images in coronal (**A**) and sagittal (**B**) plane after 6 months of healing.

Four months after bone augmentation, the clinical situation was reassessed. Due to reduced soft tissue thickness and insufficient keratinized mucosa width (Figure 2I), soft tissue augmentation using a free gingival graft (FGG) was performed (Figure 2J). After 6 months, the site was evaluated clinically (Figure 2K) and radiographically (Figure 3). Implant uncovering was performed by removing bone coronally to the implant shoulder (Figure 2L), followed by placement of a healing abutment and primary soft tissue closure (Figure 2M). A stable peri-implant soft tissue transition zone was observed four weeks later (Figure 2N).

### 3.2. Case Report 2—Surgical Treatment of Peri-Implantitis with Implant Removal

**Figure 4:**
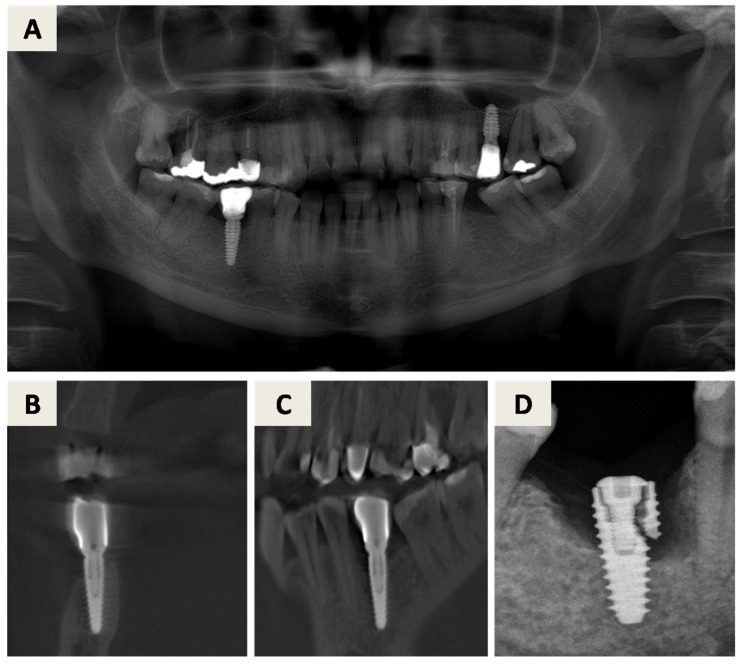
(**A**) Orthopantomogram with visible peri-implant lesion around the implant in position 46. (**B**,**C**) Cone-beam computed tomography (CBCT) images in coronal (**B**) and sagittal (**C**) planes taken in the pre-surgical phase showing a horizontal defect (Class II of the Monje classification of peri-implant bone defects); (**D**) RTG taken right before surgical procedure, demonstrating fractured implant in position 46.

**Figure 5:**
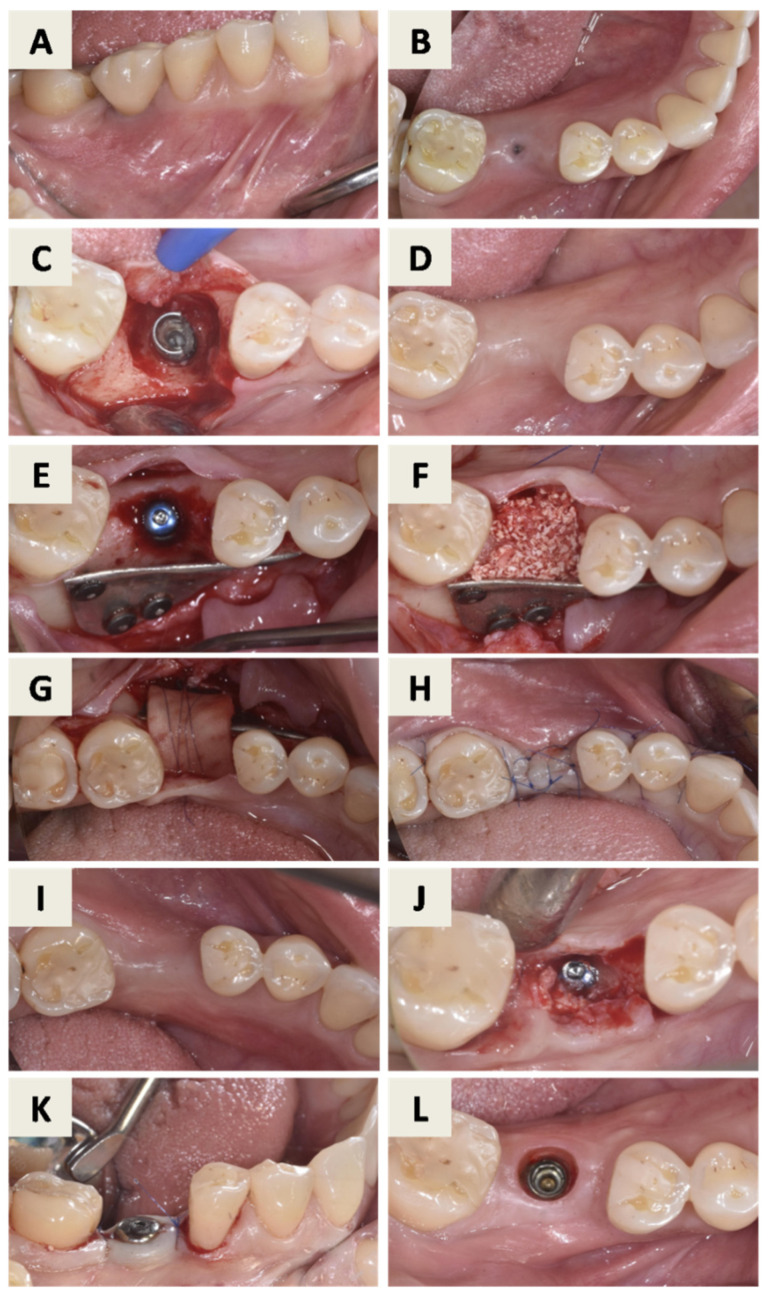
Surgical procedure: (**A**) Clinical situation before removal of the crown on the affected implant, position 46. (**B**) Healed site after 4 weeks (**C**) After the incision and the mucoperiosteal flap elevation, the fractured implant was presented and extraction was indicated. (**D**) Healed site 4 months later. (**E**) After flap elevation, the new implant is inserted. A resorbable magnesium membrane is inserted to replace the lost buccal wall with a shield technique and fixed with matching resorbable magnesium screws. (**F**) The site is filled with particles of composite bone graft consisting of autogenous bone and xenogenic bone graft. (**G**) The bone grafting particles and the magnesium membrane are covered with a dermal collagen matrix derived from porcine, which is fixed with sutures. (**H**) The flap is closed and sutured with the primary intention to heal. (**I**) Healed site after 6 months. (**J**) After the incision and the mucoperiosteal flap elevation, bone coronally from the implant shoulder had to be removed in order to expose the implant. (**K**) Primary wound closure through papilla around healing abutment. (**L**) Healed peri-implant soft tissue transition zone after 4 weeks.

Pre-surgical assessment included CBCT and orthopantomogram evaluation (Figure 4A–C) and clinical examination (Figure 5A). During the initial treatment phase, the implant-supported crown was removed, a cover screw was placed, and spontaneous soft tissue healing was allowed (Figure 5B). Prior to surgical intervention, increased suppuration was observed, prompting intraoral radiographic examination, which revealed implant fracture (Figure 4D). Consequently, implant explantation was indicated, and the treatment plan was modified to a reconstructive approach based on guided bone regeneration principles.

After flap elevation (Figure 5C), the fractured implant was carefully explanted using a trephine bur with an internal diameter of 5.0 mm. Immediate bone grafting was not performed due to active inflammation associated with the fractured implant, and delayed reconstruction was planned. Four months later, the healed site was clinically assessed (Figure 5D). A full-thickness flap was elevated via an intrasulcular incision with a vertical releasing incision at the distal aspect of tooth 45, and a new implant was placed at position 46 (Figure 5E).

A folded resorbable magnesium membrane (NOVAMag^®^ membrane, botiss biomaterials GmbH, Berlin, Germany) was inserted using the shield technique to reconstruct the missing buccal wall and fixed with resorbable magnesium screws (NOVAMag^®^ fixation screw XS, botiss biomaterials GmbH, Berlin, Germany) (Figure 5E). The defect was filled with a composite bone graft consisting of autogenous bone and bovine xenograft with hyaluronate (cerabone^®^ plus, botiss biomaterials GmbH, Berlin, Germany) (Figure 5F). The grafted area was then covered with a porcine-derived dermal collagen matrix (mucoderm^®^, botiss biomaterials GmbH, Berlin, Germany) for augmentation and fine profiling of the soft tissues and secured with sutures (Figure 5G). Primary wound closure was achieved (Figure 5H).

**Figure 6:**
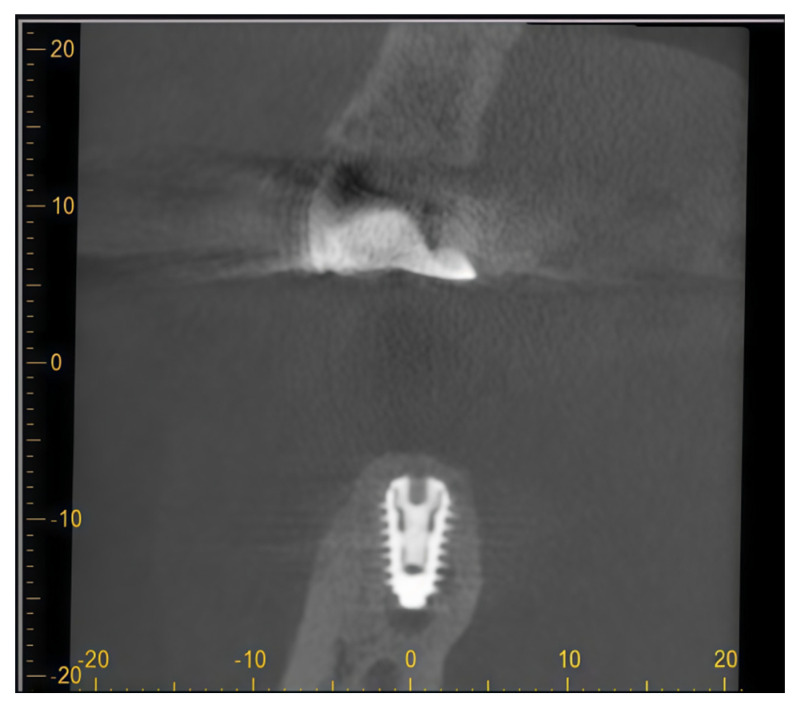
CBCT scan after 6 months of healing.

After six months, the healed site was evaluated clinically (Figure 5I). Implant uncovering was performed by removing bone coronally from the implant shoulder (Figure 5J), followed by placement of a healing abutment and primary soft tissue closure (Figure 5K). A stable peri-implant soft tissue transition zone was observed four weeks later (Figure 5L). Radiographic evaluation demonstrated favorable bone regeneration (Figure 6).

### 3.3. Case Report 3—Surgical Treatment of Peri-Implantitis Without Implant or Crown Removal

**Figure 7:**
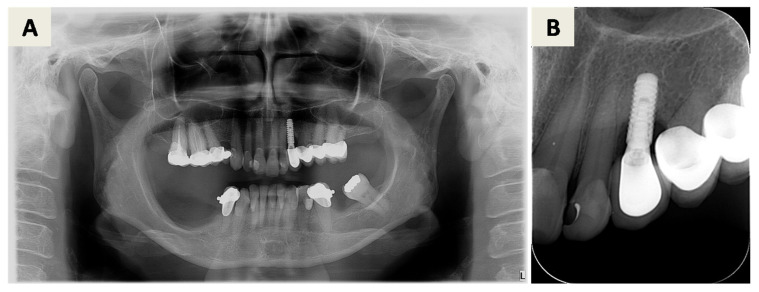
First radiographic examination. (**A**) Orthopantomogram and (**B**) RTG image showing visible peri-implantitis related bone defect around the implant in position 23 (FDI notation system).

**Figure 8:**
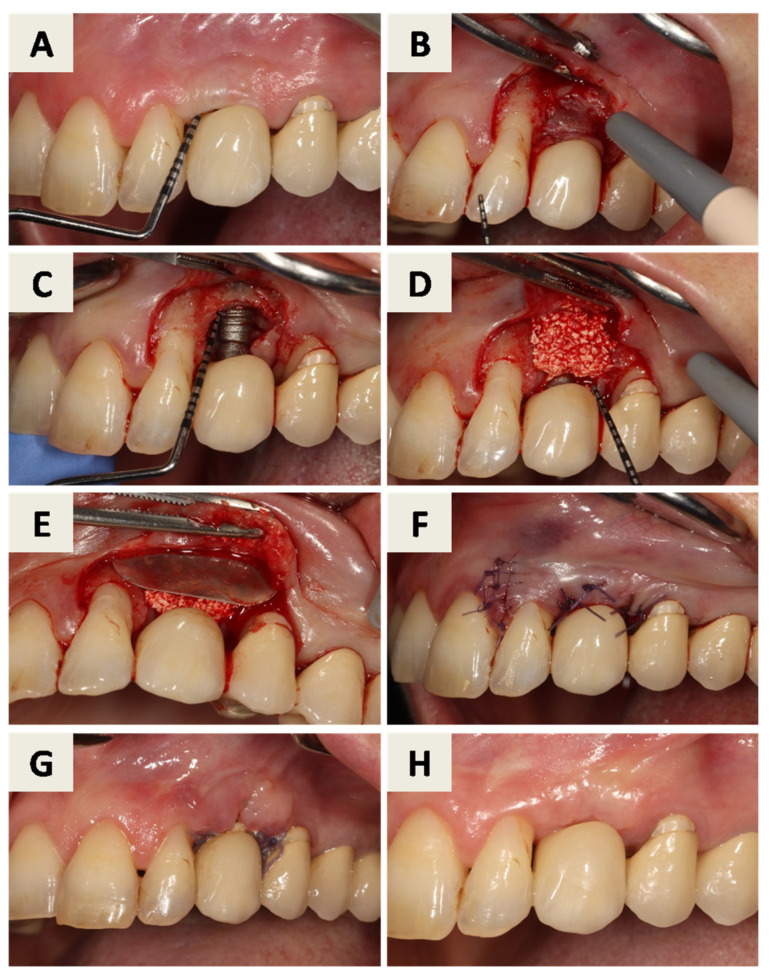
Surgical procedure: (**A**) Assessment of the clinical situation using a periodontal probe. (**B**) Full flap is elevated and granulation tissue around implant is exposed. (**C**) After mechanical debridement, the missing buccal bone is visible. (**D**) The site is filled with particles of xenogenic bone graft. (**E**) A resorbable magnesium membrane is inserted to replace the lost buccal wall with a shield technique. (**F**) The flap is closed and sutured with primary intention. (**G**) Clinical situation after 3 weeks. (**H**) Clinical situation after 6 months.

Peri-implantitis at implant position 23 was treated using a minimally invasive regenerative approach without removal of the implant or crown. Radiographic (Figure 7A,B) and pre-surgical clinical assessment (Figure 8A) revealed a peri-implant bone defect.

After surgical indication, an intrasulcular incision with one vertical releasing incision at the mesial surface of tooth 22 was performed, allowing limited elevation of a full-thickness flap to access the peri-implant defect while preserving the prosthetic restoration (Figure 8B). Granulation tissue was removed, and implant surface decontamination was performed using hand instruments and ultrasonic devices, revealing a missing buccal bone wall (Figure 8C). The defect was filled with bovine xenograft with hyaluronate (cerabone^®^ plus, botiss biomaterials GmbH, Berlin, Germany) (Figure 8D). A resorbable magnesium membrane (NOVAMag^®^ membrane, botiss biomaterials GmbH, Berlin, Germany) was adapted and inserted between the alveolar ridge and the elevated flap using the shield technique to reconstruct the buccal wall (Figure 8E). Primary wound closure was achieved (Figure 8F).

**Figure 9:**
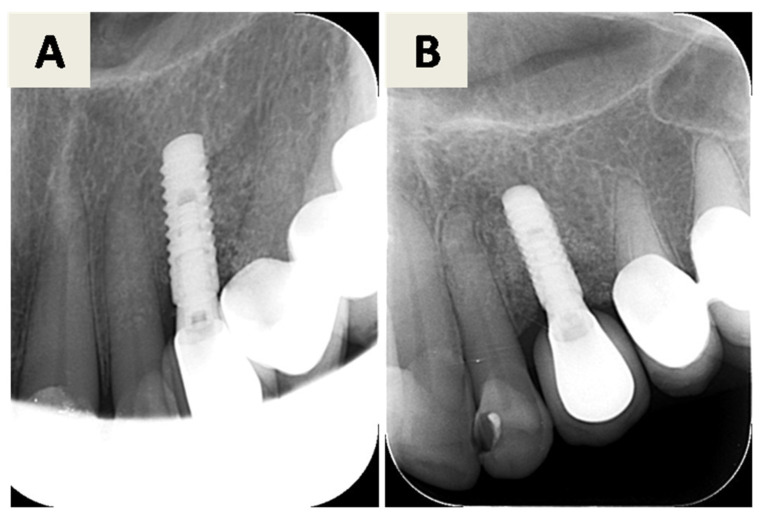
(**A**) RTG taken 3 weeks after the procedure; (**B**) RTG taken 6 months after the procedure.

Follow-up examinations demonstrated favorable soft tissue healing (Figure 8G,H) and radiographic evidence of bone regeneration (Figure 9A,B).

## 4. Therapeutic Approaches for Peri-Implantitis with Clinical and Case-Based Consideration

The treatment of peri-implantitis generally follows a stepwise approach consisting of non-surgical and, when indicated after re-evaluation, surgical interventions [16]. According to current S3-level clinical practice guidelines, the primary objectives of therapy are control of the bacterial biofilm, resolution of inflammation, and prevention of further peri-implant bone loss.

### 4.1. Non-Surgical Therapy Methods

Non-surgical therapy represents an essential initial phase in the management of peri-implantitis and aims to reduce microbial load, control inflammation, and improve patient plaque control [18,26]. This phase includes supramarginal and submarginal mechanical debridement using hand instruments, sonic or ultrasonic devices, and air-polishing systems, with particular attention to avoiding damage or roughening of the implant surface [27]. Instruments softer than titanium, such as Teflon-, carbon-, or titanium-coated curettes, are commonly recommended to minimize surface alteration and subsequent plaque retention [28].

Adjunctive use of antiseptic agents or antibiotics may be considered; however, systemic or local antibiotics should only be used as supportive measures and not as substitutes for mechanical biofilm removal [29]. Current clinical practice guidelines indicate that routine antibiotic use does not significantly improve treatment outcomes and may contribute to antimicrobial resistance, underscoring the importance of antibiotic stewardship [16].

Treatment outcomes should be re-evaluated after approximately 6–12 weeks [16]. Successful non-surgical therapy is defined by probing depths ≤5 mm, minimal bleeding on probing (≤1 site), absence of suppuration, and no further radiographic bone loss. Patients meeting these endpoints should be enrolled in a supportive peri-implant care (SPIC) program, incorporating individualized oral hygiene instruction, risk factor control, and professional mechanical plaque removal at regular intervals.

If these endpoints are not achieved, surgical intervention should be considered.

### 4.2. Surgical Therapy and Defect-Oriented Treatment Planning

Surgical treatment is indicated in cases with persistent inflammation, deep peri-implant pockets, or progressive bone loss where non-surgical measures are insufficient. Surgical therapy aims to provide direct access for thorough implant surface decontamination and, depending on defect morphology, to reduce peri-implant pockets or reconstruct lost bone support [30].

Peri-implant bone defects can be classified according to their morphology, which plays a key role in selecting the appropriate surgical approach [17]. Non-contained defects or defects with extensive horizontal bone loss generally exhibit limited regenerative potential and are therefore more suitable for resective therapy [20]. Resective procedures involve pocket elimination, apical repositioning of the flap, and often implantoplasty to reduce plaque retention on exposed implant surfaces, making them most appropriate in non-esthetic areas or in patients with low esthetic demands [31].

In contrast, contained or predominantly infraosseous defects offer greater regenerative potential and are better suited for augmentative or regenerative approaches. In cases with combined supra- and infraosseous components or partial buccal dehiscence, a combined approach involving implantoplasty of the supracrestal portion and bone augmentation of the infraosseous defect may be indicated [20].

Implant removal should be considered in advanced peri-implantitis cases depending on defect severity, implant integrity, prosthetic value, and patient-related factors [32].

### 4.3. Regenerative Surgical Concepts and Guided Bone Regeneration

Regenerative surgical therapy aims to restore lost peri-implant bone and promote re-osseointegration of the implant surface. Guided bone regeneration (GBR) is based on the use of barrier membranes to exclude rapidly proliferating soft tissue cells, stabilize the blood clot, and maintain space for bone regeneration [22].

Non-resorbable membranes, such as polytetrafluoroethylene (PTFE), provide excellent mechanical stability but require a second surgical procedure for removal and are associated with increased morbidity and risk of damage to regenerated bone [23]. To overcome these limitations, resorbable membranes have been developed, including synthetic polymers (PLA, PGA) and collagen-based membranes. Synthetic membranes may induce local acidic degradation products and inflammatory reactions [23], whereas collagen membranes, although highly biocompatible, may lack sufficient mechanical stability and collapse into the defect [24].

Resorbable magnesium membranes have recently emerged as a promising alternative, combining mechanical stability with complete biodegradability [25,33]. During degradation, magnesium releases hydroxyl ions, creating a transient alkaline environment that promotes osteogenic differentiation and osteoblast proliferation while inhibiting bacterial growth [34,35,36,37]. In addition, hydrogen gas released during degradation forms temporary gas pockets predominantly on the soft tissue–facing side of the membrane, contributing to transient soft–hard tissue separation without impairing bone formation. These properties make magnesium membranes particularly suitable for regenerative treatment in inflammatory conditions such as peri-implantitis.

### 4.4. Bone Grafting Materials and Soft Tissue Considerations

GBR procedures may involve various bone grafting materials, including autografts, allografts, xenografts, and alloplasts [22]. Autologous bone remains the gold standard due to its osteogenic, osteoinductive, and osteoconductive properties but is limited by donor-site morbidity and resorption [38]. Deproteinized bovine xenografts are widely used due to their biocompatibility, osteoconductive properties, and low resorption rate, providing long-term volume stability [39,40,41].

To enhance biological performance, bovine xenografts have been combined with hyaluronic acid or hyaluronate, which promotes angiogenesis, osteoblast activity, and bone regeneration [42,43,44]. Clinical and experimental studies have demonstrated the successful combined use of bovine xenograft with hyaluronate and resorbable magnesium membranes in regenerative procedures [45,46,47,48].

Adequate peri-implant soft tissue conditions, particularly sufficient keratinized mucosa, are essential for plaque control, inflammation reduction, and long-term peri-implant stability [49]. Insufficient keratinized tissue may increase the risk of wound dehiscence and postoperative complications following GBR, potentially compromising regenerative outcomes. Therefore, assessment and, when necessary, augmentation of peri-implant soft tissues should be considered an integral component of peri-implant regenerative therapy [50,51,52].

### 4.5. Adjunctive Decontamination Methods and Patient-Related Factors

Various adjunctive methods for implant surface decontamination have been proposed, including electrolytic cleaning systems which use electrolysis to detach biofilm from implant surfaces [53,54]. While such technologies may enhance surface decontamination, patient compliance and effective oral hygiene remain fundamental determinants of long-term treatment success.

**Figure 10:**
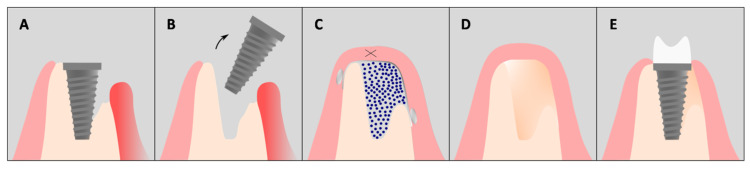
Schematic presentation of the surgical technique including implant explantation: (**A**) Implant with advanced peri-implantitis and severe bone loss. (**B**) Explantation of the unsalvageable implant. (**C**) Guided bone regeneration using bone substitute material (blue dots), magnesium membrane and magnesium fixation screws. (**D**) Regenerated bone ready for new implant placement. (**E**) Final result with new implant and prosthetic restoration.

**Figure 11:**
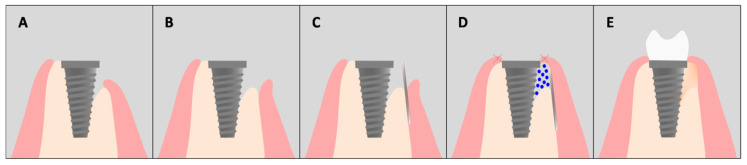
Schematic presentation of the surgical technique without implant explantation: (**A**) Implant with visible peri-implantitis. (**B**) First step is inflammation reduction by means of implant surface and soft tissue decontamination. (**C**) Defect reconstruction around implant using magnesium membrane as shield technique. (**D**) The site between implant and magnesium membrane is filled with bone substitute material (blue dots) and the flap is closed with sutures. (**E**) Final result with regenerated bone and prosthetic restoration.

In addition to established therapeutic approaches, novel biomaterials with antibacterial and pro-osteogenic properties may provide additional benefits in the management of peri-implantitis. In this context, three clinical cases have been presented in which a fully resorbable magnesium membrane was used for peri-implant bone regeneration, both with implant explantation (Figure 10) and with implant retention (Figure 11).

### 4.6. Discussion

Peri-implantitis remains a major challenge in dental implantology due to its progressive nature and potential to result in significant peri-implant bone loss and eventual implant failure if left untreated. Treatment strategies for peri-implantitis include both non-surgical and surgical approaches, which are selected based on disease severity, defect morphology, and patient-related factors. While non-surgical therapy is essential for initial infection control, surgical intervention is frequently required in advanced cases to allow adequate access to contaminated implant surfaces and reconstruction of peri-implant bone defects.

A wide range of surgical treatment modalities has been proposed for the management of peri-implantitis; however, there is currently no consensus regarding the superiority of one approach over another. The choice of treatment largely depends on the extent of bone loss and defect configuration, and a stepwise approach has been recommended to systematically address these factors [16]. In cases with advanced peri-implant bone defects, surgical therapy with flap elevation is often indicated, as it allows direct visualization of the defect, thorough debridement, and appropriate selection of resective or regenerative strategies. Recently, minimally invasive surgical approaches have been introduced, aiming to reduce patient morbidity and soft tissue recession while maintaining acceptable clinical outcomes [55]. These approaches may represent a viable alternative in selected cases and offer the advantage of reduced postoperative discomfort and shorter recovery time.

Regenerative surgical therapy aims to restore lost peri-implant bone support and promote re-osseointegration of the affected implant surface. Guided bone regeneration (GBR) has therefore become a cornerstone in the management of peri-implant bone defects with favorable morphology. Nevertheless, the predictability of GBR is influenced by several factors, including defect configuration, implant surface decontamination, peri-implant soft tissue conditions, and the biological and mechanical properties of the materials used [21].

In this context, the use of a magnesium membrane represents an innovative approach for guided bone regeneration due to its unique combination of biodegradability, mechanical stability, and biocompatibility [56]. Unlike conventional resorbable membranes, magnesium membranes provide sufficient rigidity to maintain space over the defect while being fully resorbed, thereby eliminating the need for a second surgical procedure for membrane removal. This mechanical stability is particularly relevant in larger or non-self-supporting defects, where maintenance of the regenerative space is critical [57].

Beyond their barrier function, magnesium membranes exhibit bioactive properties that may further support regenerative outcomes. During degradation, magnesium releases hydroxyl ions, creating a transient alkaline environment that promotes osteogenic differentiation of mesenchymal stem cells and enhances osteoblast proliferation. In addition, alkaline conditions have been shown to inhibit bacterial growth by interfering with microbial metabolism and inducing oxidative stress, as well as reducing bacterial adhesion and preventing biofilm formation [58]. Another characteristic of magnesium degradation is the release of hydrogen gas, which forms transient gas pockets predominantly on the soft tissue–facing side of the membrane. These gas pockets contribute to temporary separation between soft and hard tissues without impairing bone formation, thereby supporting the principles of guided bone regeneration [25]. Collectively, these properties suggest that magnesium membranes may function not only as passive barriers but also as adjunctive modulators of the regenerative environment, which is particularly relevant in inflammatory conditions such as peri-implantitis.

The clinical use of magnesium membranes has already been documented for several indications, including direct and indirect sinus floor elevation procedures [59], shield techniques for ridge preservation and immediate implant placement in the esthetic zone [57,60], and the regeneration of periodontal intrabony defects [33]. In addition, the successful simultaneous use of magnesium membranes and bovine xenografts containing hyaluronate has been reported, as well as in vitro evidence demonstrating that the addition of hyaluronic acid does not adversely affect magnesium membrane degradation [48]. The present case series further supports these findings by demonstrating the clinical feasibility of magnesium membranes in different peri-implantitis treatment scenarios, including cases with implant retention, implant removal, and minimally invasive regenerative approaches.

Bone graft selection represents another critical factor influencing regenerative outcomes. Autologous bone remains the gold standard for bone augmentation due to its osteogenic, osteoinductive, and osteoconductive properties; however, its use is limited by donor-site morbidity and physiologically high resorption rates. Deproteinized bovine bone is widely used as an alternative due to its favorable osteoconductive properties and low resorption rate, providing long-term volume stability. To enhance its biological performance, bovine xenografts have been combined with hyaluronic acid or hyaluronate, which has been shown to promote angiogenesis, osteoblast activity, and bone formation.

Experimental and clinical studies have highlighted the positive effects of hyaluronate in periodontal and implant-related regenerative procedures [61,62]. Clinical trials have demonstrated improved defect filling and long-term stability when bovine xenografts are used in regenerative peri-implant therapy, particularly when combined with barrier membranes. Additional benefits of hyaluronic acid include antimicrobial and anti-inflammatory effects, as well as modulation of proinflammatory cytokines such as IL-1β, which may further support a favorable healing environment [63,64,65].

Peri-implant soft tissue conditions play a crucial role in the success of regenerative therapy. Adequate keratinized mucosa has been associated with improved plaque control, reduced peri-implant inflammation, and greater clinical stability around dental implants [49]. Conversely, insufficient keratinized tissue may increase the risk of wound dehiscence and postoperative complications following guided bone regeneration, potentially compromising regenerative outcomes [51]. Therefore, careful assessment of peri-implant soft tissues and soft tissue augmentation, when indicated, should be considered integral components of peri-implantitis treatment planning [50,52].

Although the present case series demonstrates favorable clinical and radiographic outcomes using a resorbable magnesium membrane in combination with bovine xenograft containing hyaluronate, several limitations must be acknowledged. The small number of cases and the absence of a control group limit the generalizability of the findings. Future prospective studies with larger sample sizes, standardized defect classifications, and long-term follow-up are needed to further evaluate the clinical effectiveness and long-term stability of this regenerative approach.

## 5. Conclusions

Peri-implantitis requires a multifaceted treatment approach tailored to disease severity and defect morphology. While non-surgical therapy is essential for initial infection control, advanced cases often require surgical intervention and regenerative strategies. The use of guided bone regeneration remains a cornerstone for peri-implant bone reconstruction.

Within this context, resorbable magnesium membranes represent a promising alternative to conventional barrier membranes by combining mechanical stability, complete biodegradability, and bioactive properties that may support osteogenesis and indirectly modulate bacterial activity. The presented clinical cases illustrate the potential applicability of this novel membrane in different peri-implantitis scenarios. Further controlled clinical studies are required to confirm its long-term effectiveness and to define its role within contemporary peri-implant regenerative therapy.

## Data Availability

The original contributions presented in this study are included in the article. Further inquiries can be directed to the corresponding author.

## References

[1] Dierens M., Vandeweghe S., Kisch J., Persson G.R., Cosyn J., De Bruyn H. (2013). Long-Term Follow-Up of Turned Single Implants Placed in Periodontally Healthy Patients After 16 to 22 Years: Microbiologic Outcome. J. Periodontol..

[2] Howe M.S., Keys W., Richards D. (2019). Long-Term (10-Year) Dental Implant Survival: A Systematic Review and Sensitivity Meta-Analysis. J. Dent..

[3] Karlsson K., Derks J., Wennström J.L., Petzold M., Berglundh T. (2020). Occurrence and Clustering of Complications in Implant Dentistry. Clin. Oral Implant. Res..

[4] Berglundh T., Persson L., Klinge B. (2002). A Systematic Review of the Incidence of Biological and Technical Complications in Implant Dentistry Reported in Prospective Longitudinal Studies of at Least 5 Years. J. Clin. Periodontol..

[5] Berglundh T., Armitage G., Araujo M.G., Avila-Ortiz G., Blanco J., Camargo P.M., Chen S., Cochran D., Derks J., Figuero E. (2018). Peri-Implant Diseases and Conditions: Consensus Report of Workgroup 4 of the 2017 World Workshop on the Classification of Periodontal and Peri-Implant Diseases and Conditions. J. Clin. Periodontol..

[6] Renvert S., Persson G.R., Pirih F.Q., Camargo P.M. (2018). Peri-Implant Health, Peri-Implant Mucositis, and Peri-Implantitis: Case Definitions and Diagnostic Considerations. J. Periodontol..

[7] Berglundh T., Jepsen S., Stadlinger B., Terheyden H. (2019). Peri-Implantitis and Its Prevention. Clin. Oral Implant. Res..

[8] Schwarz F., Derks J., Monje A., Wang H.L. (2018). Peri-Implantitis. J. Clin. Periodontol..

[9] Berglundh T., Mombelli A., Schwarz F., Derks J. (2024). Etiology, Pathogenesis and Treatment of Peri-Implantitis: A European Perspective. Periodontol. 2000.

[10] Darby I. (2022). Risk Factors for Periodontitis & Peri-Implantitis. Periodontol. 2000.

[11] Berglundh T., Zitzmann N.U., Donati M. (2011). Are Peri-Implantitis Lesions Different from Periodontitis Lesions?. J. Clin. Periodontol..

[12] Cortelli S.C., Cortelli J.R., Romeiro R.L., Costa F.O., Aquino D.R., Orzechowski P.R., Araújo V.C., Duarte P.M. (2013). Frequency of Periodontal Pathogens in Equivalent Peri-Implant and Periodontal Clinical Statuses. Arch. Oral Biol..

[13] Carvalho É.B.S., Romandini M., Sadilina S., Sant’Ana A.C.P., Sanz M. (2023). Microbiota Associated with Peri-implantitis—A Systematic Review with Meta-Analyses. Clin. Oral Implant. Res..

[14] Persson G.R., Renvert S. (2014). Cluster of Bacteria Associated with Peri-Implantitis. Clin. Implant Dent. Relat. Res..

[15] Faot F., Nascimento G.G., Bielemann A.M., Campão T.D., Leite F.R.M., Quirynen M. (2015). Can Peri-Implant Crevicular Fluid Assist in the Diagnosis of Peri-Implantitis? A Systematic Review and Meta-Analysis. J. Periodontol..

[16] Herrera D., Berglundh T., Schwarz F., Chapple I., Jepsen S., Sculean A., Kebschull M., Papapanou P.N., Tonetti M.S., Sanz M. (2023). Prevention and Treatment of Peri-Implant Diseases-The EFP S3 Level Clinical Practice Guideline. J. Clin. Periodontol..

[17] Monje A., Pons R., Insua A., Nart J., Wang H.L., Schwarz F. (2019). Morphology and Severity of Peri-Implantitis Bone Defects. Clin. Implant Dent. Relat. Res..

[18] Wang C.W., Renvert S., Wang H.L. (2019). Nonsurgical Treatment of Periimplantitis. Implant Dent..

[19] Schwarz F., Jepsen S., Obreja K., Galarraga-Vinueza M.E., Ramanauskaite A. (2022). Surgical Therapy of Peri-Implantitis. Periodontol. 2000.

[20] Monje A., Schwarz F. (2022). Principles of Combined Surgical Therapy for the Management of Peri-Implantitis. Clin. Adv. Periodontics.

[21] Monje A., Pons R., Nart J., Miron R.J., Schwarz F., Sculean A. (2023). Selecting Biomaterials in the Reconstructive Therapy of Peri-Implantitis. Periodontol. 2000.

[22] Liu J., Kerns D.G. (2014). Mechanisms of Guided Bone Regeneration: A Review. Open Dent. J..

[23] Gentile P., Chiono V., Tonda-Turo C., Ferreira A.M., Ciardelli G. (2011). Polymeric Membranes for Guided Bone Regeneration. Biotechnol. J..

[24] Ren Y., Fan L., Alkildani S., Liu L., Emmert S., Najman S., Rimashevskiy D., Schnettler R., Jung O., Xiong X. (2022). Barrier Membranes for Guided Bone Regeneration (GBR): A Focus on Recent Advances in Collagen Membranes. Int. J. Mol. Sci..

[25] Rider P., Kačarević Ž.P., Elad A., Tadic D., Rothamel D., Sauer G., Bornert F., Windisch P., Hangyási D.B., Molnar B. (2022). Biodegradable Magnesium Barrier Membrane Used for Guided Bone Regeneration in Dental Surgery. Bioact. Mater..

[26] Figuero E., Graziani F., Sanz I., Herrera D., Sanz M. (2014). Management of Peri-Implant Mucositis and Peri-Implantitis. Periodontol. 2000.

[27] Unursaikhan O., Lee J.S., Cha J.K., Park J.C., Jung U.W., Kim C.S., Cho K.S., Choi S.H. (2012). Comparative Evaluation of Roughness of Titanium Surfaces Treated by Different Hygiene Instruments. J. Periodontal Implant Sci..

[28] Valderrama P., Wilson T.G. (2013). Detoxification of Implant Surfaces Affected by Peri-Implant Disease: An Overview of Surgical Methods. Int. J. Dent..

[29] Smeets R., Henningsen A., Jung O., Heiland M., Hammächer C., Stein J.M. (2014). Definition, Etiology, Prevention and Treatment of Peri-Implantitis—A Review. Head Face Med..

[30] Khoury F., Keeve P.L., Ramanauskaite A., Schwarz F., Koo K.T., Sculean A., Romanos G. (2019). Surgical Treatment of Peri-Implantitis—Consensus Report of Working Group 4. Int. Dent. J..

[31] Monje A., Pons R., Amerio E., Wang H.L., Nart J. (2022). Resolution of Peri-Implantitis by Means of Implantoplasty as Adjunct to Surgical Therapy: A Retrospective Study. J. Periodontol..

[32] Ramanauskaite A., Schwarz F. (2024). Current Concepts for the Treatment of Peri-Implant Disease. Int. J. Prosthodont..

[33] Hangyasi D.B., Körtvélyessy G., Blašković M., Rider P., Rogge S., Siber S., Kačarević Ž.P., Čandrlić M. (2023). Regeneration of Intrabony Defects Using a Novel Magnesium Membrane. Medicina.

[34] Rider P., Kačarević Ž.P., Elad A., Rothamel D., Sauer G., Bornert F., Windisch P., Hangyási D., Molnar B., Hesse B. (2022). Analysis of a Pure Magnesium Membrane Degradation Process and Its Functionality When Used in a Guided Bone Regeneration Model in Beagle Dogs. Materials.

[35] Wang C.-X., Ma T., Wang M.-Y., Guo H.-Z., Ge X.-Y., Zhang Y., Lin Y. (2021). Facile Distribution of an Alkaline Microenvironment Improves Human Bone Marrow Mesenchymal Stem Cell Osteogenesis on a Titanium Surface through the ITG/FAK/ALP Pathway. Int. J. Implant Dent..

[36] Kačarević Ž.P., Rider P., Elad A., Tadic D., Rothamel D., Sauer G., Bornert F., Windisch P., Hangyási D.B., Molnar B. (2022). Biodegradable Magnesium Fixation Screw for Barrier Membranes Used in Guided Bone Regeneration. Bioact. Mater..

[37] Tan J., Wang D., Cao H., Qiao Y., Zhu H., Liu X. (2018). Effect of Local Alkaline Microenvironment on the Behaviors of Bacteria and Osteogenic Cells. ACS Appl. Mater. Interfaces.

[38] Zhao R., Yang R., Cooper P.R., Khurshid Z., Shavandi A., Ratnayake J. (2021). Bone Grafts and Substitutes in Dentistry: A Review of Current Trends and Developments. Molecules.

[39] Perić Kačarević Ž., Rider P., Alkildani S., Retnasingh S., Pejakić M., Schnettler R., Gosau M., Smeets R., Jung O., Barbeck M. (2020). An Introduction to Bone Tissue Engineering. Int. J. Artif. Organs.

[40] Jensen S.S., Broggini N., Hjørting-Hansen E., Schenk R., Buser D. (2006). Bone Healing and Graft Resorption of Autograft, Anorganic Bovine Bone and β-Tricalcium Phosphate. A Histologic and Histomorphometric Study in the Mandibles of Minipigs. Clin. Oral Implant. Res..

[41] Accorsi-Mendonça T., Conz M.B., Barros T.C., de Sena L.Á., Soares G.d.A., Granjeiro J.M. (2008). Physicochemical Characterization of Two Deproteinized Bovine Xenografts. Braz. Oral Res..

[42] Fraser J.R.E., Laurent T.C., Laurent U.B.G. (1997). Hyaluronan: Its Nature, Distribution, Functions and Turnover. J. Intern. Med..

[43] Kyyak S., Blatt S., Wiesmann N., Smeets R., Kaemmerer P.W. (2022). Hyaluronic Acid with Bone Substitutes Enhance Angiogenesis In Vivo. Materials.

[44] Kyyak S., Pabst A., Heimes D., Kämmerer P.W., Weber F.E. (2021). Materials The Influence of Hyaluronic Acid Biofunctionalization of a Bovine Bone Substitute on Osteoblast Activity In Vitro. Materials.

[45] Arpağ O.F., Damlar İ., Altan A., Tatli U., Günay A. (2018). To What Extent Does Hyaluronic Acid Affect Healing of Xenografts? A Histomorphometric Study in a Rabbit Model. J. Appl. Oral Sci..

[46] Lee J.B., Chu S., Ben Amara H., Song H.Y., Son M.J., Lee J., Kim H.Y., Koo K.T., Rhyu I.C. (2021). Effects of Hyaluronic Acid and Deproteinized Bovine Bone Mineral with 10% Collagen for Ridge Preservation in Compromised Extraction Sockets. J. Periodontol..

[47] Kim J.-J., Song H.Y., Ben Amara H., Kyung-Rim K., Koo K.-T. (2016). Hyaluronic Acid Improves Bone Formation in Extraction Sockets With Chronic Pathology: A Pilot Study in Dogs. J. Periodontol..

[48] Blašković M., Blašković D., Hangyasi D.B., Peloza O.C., Tomas M., Čandrlić M., Rider P., Mang B., Kačarević Ž.P., Trajkovski B. (2023). Evaluation between Biodegradable Magnesium Metal GBR Membrane and Bovine Graft with or without Hyaluronate. Membranes.

[49] Zhang Z., Zhang Z., Wang P., Zheng Y., Wang Z., Wang Z. (2025). The Relationship between Adequate Keratinized Mucosa and Peri-Implant Disease: A Systematic Review and Meta-Analysis. BMC Oral Health.

[50] Wang J., Xie C., Wei H., Yu Z., Li D. (2025). Effectiveness of Keratinized Mucosa Augmentation Procedures around Dental Implants Based on Risk Assessment: A 5-Year Retrospective Cohort Study. J. Prosthodont. Res..

[51] Roccuzzo A., Imber J.C., Stähli A., Romandini M., Sculean A., Salvi G.E., Roccuzzo M. (2025). Role of Keratinized Mucosa on the Risk of Peri-Implant Diseases and Soft Tissue Dehiscence in the Posterior Mandible—A 20-Year Prospective Cohort Study. J. Periodontal Res..

[52] Zhang S., Sheng R., Fan Z., Wang F., Di P., Shi J., Zou D., Li D., Zhang Y., Chen Z. (2025). Expert Consensus on Peri-Implant Keratinized Mucosa Augmentation at Second-Stage Surgery. Int. J. Oral Sci..

[53] Schlee M., Rathe F., Brodbeck U., Ratka C., Weigl P., Zipprich H. (2019). Treatment of Peri-Implantitis-Electrolytic Cleaning versus Mechanical and Electrolytic Cleaning- A Randomized Controlled Clinical Trial- Six-Month Results. J. Clin. Med..

[54] Ratka C., Weigl P., Henrich D., Koch F., Schlee M., Zipprich H. (2019). The Effect of in Vitro Electrolytic Cleaning on Biofilm-Contaminated Implant Surfaces. J. Clin. Med..

[55] Carrillo de Albornoz A., Montero E., Alonso-Español A., Sanz M., Sanz-Sánchez I. (2024). Treatment of Peri-Implantitis with a Flapless Surgical Access Combined with Implant Surface Decontamination and Adjunctive Systemic Antibiotics: A Retrospective Case Series Study. J. Clin. Periodontol..

[56] Zhang T., Wang W., Liu J., Wang L., Tang Y., Wang K. (2022). A Review on Magnesium Alloys for Biomedical Applications. Front. Bioeng. Biotechnol..

[57] Elad A., Rider P., Rogge S., Witte F., Tadić D., Kačarević Ž.P., Steigmann L. (2023). Application of Biodegradable Magnesium Membrane Shield Technique for Immediate Dentoalveolar Bone Regeneration. Biomedicines.

[58] Li Y., Liu G., Zhai Z., Liu L., Li H., Yang K., Tan L., Wan P., Liu X., Ouyang Z. (2014). Antibacterial Properties of Magnesium in Vitro and in an in Vivo Model of Implant-Associated Methicillin-Resistant Staphylococcus Aureus Infection. Antimicrob. Agents Chemother..

[59] Elad A., Pul L., Rider P., Rogge S., Witte F., Tadić D., Mijiritsky E., Kačarević Ž.P., Steigmann L. (2023). Resorbable Magnesium Metal Membrane for Sinus Lift Procedures: A Case Series. BMC Oral Health.

[60] Blašković M., Butorac Prpić I., Aslan S., Gabrić D., Blašković D., Cvijanović Peloza O., Čandrlić M., Perić Kačarević Ž. (2024). Magnesium Membrane Shield Technique for Alveolar Ridge Preservation: Step-by-Step Representative Case Report of Buccal Bone Wall Dehiscence with Clinical and Histological Evaluations. Biomedicines.

[61] Božić D., Ćatović I., Badovinac A., Musić L., Par M., Sculean A. (2021). Treatment of Intrabony Defects with a Combination of Hyaluronic Acid and Deproteinized Porcine Bone Mineral. Materials.

[62] Rakašević D., Šćepanović M., Mijailović I., Mišić T., Janjić B., Soldatović I., Marković A. (2023). Reconstructive Peri-Implantitis Therapy by Using Bovine Bone Substitute with or without Hyaluronic Acid: A Randomized Clinical Controlled Pilot Study. J. Funct. Biomater..

[63] Kawano M., Ariyoshi W., Iwanaga K., Okinaga T., Habu M., Yoshioka I., Tominaga K., Nishihara T. (2011). Mechanism Involved in Enhancement of Osteoblast Differentiation by Hyaluronic Acid. Biochem. Biophys. Res. Commun..

[64] Sánchez-Fernández E., Magán-Fernández A., O’Valle F., Bravo M., Mesa F. (2021). Hyaluronic Acid Reduces Inflammation and Crevicular Fluid IL-1β Concentrations in Peri-Implantitis: A Randomized Controlled Clinical Trial. J. Periodontal Implant Sci..

[65] Soriano-Lerma A., Magán-Fernández A., Gijón J., Sánchez-Fernández E., Soriano M., García-Salcedo J.A., Mesa F. (2020). Short-Term Effects of Hyaluronic Acid on the Subgingival Microbiome in Peri-Implantitis: A Randomized Controlled Clinical Trial. J. Periodontol..

